# Structural and Enzymological Evidence for an Altered Substrate Specificity in Okur-Chung Neurodevelopmental Syndrome Mutant CK2α^Lys198Arg^

**DOI:** 10.3389/fmolb.2022.831693

**Published:** 2022-04-04

**Authors:** Christian Werner, Alexander Gast, Dirk Lindenblatt, Anna Nickelsen, Karsten Niefind, Joachim Jose, Jennifer Hochscherf

**Affiliations:** ^1^ Department of Chemistry, Institute of Biochemistry, University of Cologne, Cologne, Germany; ^2^ Institute of Pharmaceutical and Medicinal Chemistry, University of Münster, Münster, Germany

**Keywords:** okur-chung neurodevelopmental syndrome (OCNDS), protein kinase CK2, casein kinase 2, CSNK2A1 gene, acidophilic substrate specificity, substrate recognition, P+1 loop, anion binding site

## Abstract

Specific *de novo* mutations in the *CSNK2A1* gene, which encodes CK2α, the catalytic subunit of protein kinase CK2, are considered as causative for the Okur-Chung neurodevelopmental syndrome (OCNDS). OCNDS is a rare congenital disease with a high phenotypic diversity ranging from neurodevelopmental disabilities to multi-systemic problems and characteristic facial features. A frequent OCNDS mutation is the exchange of Lys198 to Arg at the center of CK2α′s P+1 loop, a key element of substrate recognition. According to preliminary data recently made available, this mutation causes a significant shift of the substrate specificity of the enzyme. We expressed the CK2α^Lys198Arg^ recombinantly and characterized it biophysically and structurally. Using isothermal titration calorimetry (ITC), fluorescence quenching and differential scanning fluorimetry (Thermofluor), we found that the mutation does not affect the interaction with CK2β, the non-catalytic CK2 subunit, and that the thermal stability of the protein is even slightly increased. However, a CK2α^Lys198Arg^ crystal structure and its comparison with wild-type structures revealed a significant shift of the anion binding site harboured by the P+1 loop. This observation supports the notion that the Lys198Arg mutation causes an alteration of substrate specificity which we underpinned here with enzymological data.

## Introduction

The serine/threonine kinase CK2, a member of the CMGC branch within the superfamily of eukaryotic protein kinases (EPKs) (Manning et al., 2002), is a heterotetrametric holoenzyme consisting of a central dimer of non-catalytic subunits (CK2β) to which two separate catalytic subunits (CK2α or CK2α’ encoded in *Homo sapiens* by the genes CSNK2A1 or CSNK2A2, respectively) are attached (Niefind et al., 2001). Unlike many other EPKs—in particular its closest relatives within the CMGC family—, CK2 it is constitutively active (Niefind et al., 2007) in the sense that it does not require a phosphorylation or any other posttranslational modification for activation; alternatively, salt- and polycation-dependent self-interactions of CK2α_2_β_2_ holoenzyme complexes were proposed as a regulatory mechanism (Niefind and Issinger, 2005; Poole et al., 2005; Schnitzler et al., 2014) and experimental evidence of such interactions in the cell was provided (Hübner et al., 2014).

CK2 is involved in important cellular processes such as pro-survival signaling (St-Denis and Litchfield, 2009; Zheng et al., 2013; Castello et al., 2017), cell proliferation (Lebrin et al., 2001; St-Denis and Litchfield, 2009) and DNA-damage repair (Loizou et al., 2004; Montenarh, 2016). CK2 is extremely pleiotropic with more than 300 protein substrates reported already in 2003 (Meggio and Pinna, 2003). From these substrates as well as from peptide studies (Marchiori et al., 1988; Sarno et al., 1997), the consensus sequence S/T-D/E-X-D/E for CK2 substrate recognition was derived. The extraordinary pleiotropy of CK2 was emphasized later by Salvi et al. (2009) who extracted 2,275 potential CK2 sites out of 10,899 phospho sites altogether in a database analysis and validated some of the newly detected CK2 sites experimentally. In a review of phosphoproteomic studies, Needham at al. (2019) collected even 671 substrate proteins of CK2α or CK2α’, 445 of them being functionally annotated.

CK2 is expressed in various tissues (Faust and Montenarh, 2000) and particularly highly in the mammalian brain (Blanquet, 2000; Castello et al., 2017). Fitting to this, it was linked to neurodegenerative disorders such as Parkinson’s disease (Ryu et al., 2008; Borgo et al., 2021) and Alzheimer’s disease (Greenwood et al., 1994; Rosenberger et al., 2016) as well as to brain tumours like glioblastoma (Nitta et al., 2015; Rowse et al., 2017). The Okur-Chung neurodevelopmental syndrome (OCNDS) is a recent addition to CK2-associated pathologies of the nervous system. OCNDS is a rare disease observed predominantly in children and affects the patients’ behavior, facial structures, physical, intellectual and psychological development as well as their overall health status (Okur et al., 2016; Trinh et al., 2017; Akahira-Azuma et al., 2018; Chiu et al., 2018; Owen et al., 2018; Nakashima et al., 2019; Xu et al., 2020). Currently, about 120 OCNDS patients are known worldwide (https:/www.csnk2a1foundation.org/, retrieved at 21^st^ of June 2021).

OCNDS is considered as linked to *de novo* mutations in one allele of the *CSNK2A1* gene as revealed by whole exome sequencing (Okur et al., 2016; Akahira-Azuma et al., 2018; Chiu et al., 2018; Owen et al., 2018; Nakashima et al., 2019; Xu et al., 2020). In a recent review, 35 different *CSNK2A1* mutations relevant for OCNDS are listed (Wu et al., 2021), among them the most common one, which is Lys198Arg (Nakashima et al., 2019). Lys198 is located in the P+1 loop, the C-terminal part of the activation segment. The P+1 loop is typically used in EPKs for substrate recognition. In CK2α, Lys198 plus two further basic residues (Arg191 and Arg195) impart a positively charged character to the P+1 loop fitting to the enzyme’s preference for a negatively charged side chain one position downstream from the phosphorylated substrate residue. This functionality of CK2α′s P+1 loop was disclosed by a mutational study (Sarno et al., 1997); its structural basis was revealed by crystal structures of human CK2α with two sulfate ions (one of them harboured at the P+1 loop) (Niefind et al., 2007) and with the polyanionic substrate-competitive inhibitor heparin (Schnitzler and Niefind, 2021).

Typically, the CK2α mutations occurring in OCNDS are mentioned in clinical reports together with phenotypic features (Trinh et al., 2017; Akahira-Azuma et al., 2018; Chiu et al., 2018; Owen et al., 2018; Xu et al., 2020), while investigations on the protein level are rare so far. Recently, Dominguez et al. (2021) observed a general loss of catalytic activity for 15 OCNDS-found CK2α mutants (among them CK2α^Lys198Arg^) expressed as GST-fusion proteins in bacteria, however, only with a standard peptide substrate and without an enzymologically stringent distinction in K_M_- and k_cat_-values. Caefer et al. (2021) performed a sophisticated phosphoproteomics study in a bacterial system (Chou et al., 2012; Lubner et al., 2018) and published preliminary evidence that the critical effect of the Lys198Arg mutation might concern the substrate specificity of CK2α rather than the overall activity. To supplement these data and to attempt to resolve the discrepancy, we report here the results of a crystallographic, biophysical and enzymological investigation of the Lys198Arg mutant of CK2α.

## Materials and Methods

### Preparation of CK2α and CK2β Variants

Synthetic genes for human CK2α, CK2α^1-335^, CK2α^Lys198Arg^, and CK2α^1-335,Lys198Arg^ embedded in pET-28a (+) plasmids and thus prepared to carry an N-terminal (His)_6_-tag were purchased from BioCat, Heidelberg; here, CK2α^1-335^ and CK2α^1-335,Lys198Arg^ were included as backups because CK2α^1-335^—in contrast to the full-length enzyme—does not tend to degrade C-terminally (Niefind et al., 2000), but it is functionally fully competent with respect to catalytic activity (Ermakova et al., 2003) and interaction with CK2β (Raaf et al., 2008). Competent *Escherichia coli* BL21 (DE3) cells were transformed with those plasmids and plated onto agar plates containing 100 μg/ml kanamycin. Grown colonies were picked and used for precultures containing 100 ml of lysogeny broth (LB) medium (10 g/L yeast extract, 20 g/L tryptone, 20 g/L sodium chloride) endowed with 100 μg/ml kanamycin. The precultures were grown over night at 37°C under shaking at 180 rpm. They were used to inoculate main cultures of 5 L slightly modified terrific broth medium (10 g/L yeast extract, 20 g/L tryptone, 2 g/L potassium dihydrogen phosphate, 8 g/L di-potassium hydrogen phosphate and 0.4 g/L magnesium sulfate) supplemented with 100 μg/ml kanamycin.

Each main culture was shaken at 37°C with 180 rpm until an OD_600_ between 1.0 and 1.5 was reached. Then, gene expression was induced by adding IPTG (Anatrace) to a final concentration of 0.5 mM. After incubating overnight at 20°C, the cells were harvested by centrifugation at 6,200 x g and 4°C for 30 min. The resulting cell pellets were frozen at −80°C after washing once with 0.9% (w/v) NaCl. The cells were lysed by incubation in lysis buffer (500 mM NaCl, 25 mM TRIS/HCl, pH 8.5, 1 mg/ml lysozyme and 10 μg/ml DNaseI) for 30 min at 4°C and subsequent cautious sonification (2 min, 2 s on/2 s off, 4°C, 40% power). The cell lysate was centrifuged at 186,000 x g and 4°C for 30 min to remove cell debris. The supernatant was filtered and then applied onto Ni-NTA affinity chromatography column (HisTrap™ FF 5 ml, GE Healthcare) mounted on an ÄKTA Prime chromatography system. The buffer A for sample application and washing was composed of 500 mM NaCl, 25 mM TRIS/HCl, pH 8.5 and 40 mM imidazole while the buffer B for gradient elution contained 250 mM rather than 40 mM imidazole. The protein was eluted using a linear gradient of 15 column volumes. Fractions with high absorption at 280 nm were analyzed more precisely by TRIS-glycine SDS-PAGE; those containing the desired protein were pooled and then concentrated by ultrafiltration using AMICON® ultra tubes (cutoff 30 kDa). Simultaneously, the protein was rebuffered into its standard background and storage buffer (500 mM NaCl, 25 mM TRIS/HCl, pH 8.5).

Human CK2β was prepared as full-length protein and as a C-terminal truncation construct CK2β^1-193^ which can interact with CK2α to form a CK2α_2_β_2_ holoenzyme (Boldyreff et al., 1993; Raaf et al., 2008). The pT7-7 plasmid containing the gene for CK2β^1–193^ without any tag was transformed into *Escherichia coli* BL21 (DE3) cells. The expression, harvesting and lysing procedure was analogue to the CK2α variants with the exception that LB medium with 100 μg/ml ampicillin was used for the pre- and main culture and that at an OD_600_ of 0.7, IPTG was added to a final concentration of 0.5 mM. Recombinant CK2β^1-193^ was prepared with a two-step chromatographic protocol: anion exchange chromatography on a HiTrap Sepharose Q column (GE HealthCare) followed by affinity chromatography with a heparin column (GE HealthCare). For both chromatographic steps, the low-salt buffer contained 150 mM NaCl 25 mM TRIS/HCl, pH 8.5 and the high-salt buffer contained 1 M NaCl 25 mM TRIS/HCl, pH 8.5. The protocol for the chromatography, analysis of the fractions and rebuffering was analogous to the CK2α variants.

To determine enzymatic parameters, CK2α_2_β_2_ holoenzyme variants consisting of either CK2α or CK2α^Lys198Arg^ in complex with CK2β^1-193^ were prepared. For this, the mentioned pET-28a (+) vector with the CK2α and CK2α^Lys198Arg^ genes as well as a pT7-7 vector with the coding sequence for CK2β^1-193^ with an N-terminal (His)_6_-tag were used. Transformation of competent *Escherichia coli* BL21 (DE3) cells, expression, harvesting, and cell lysis were performed in a similar manner as described above. For the expression of CK2β^1-193^, ampicillin was substituted for carbenicillin as selection marker in bacterial culture. LB agar plates, 50 ml LB medium precultures and 0.5 L LB medium main cultures contained 50 μg/ml kanamycin or carbenicillin.

Gene expression was induced at an OD_578_ between 0.5 and 0.6 by adding IPTG to a final concentration of 1 mM. After incubation for 4 h at 30°C, cells were harvested by centrifugation at 5,000 x g and 4°C for 10 min. To constitute the respective CK2α_2_β_2_ holoenzyme variants upon cell lysis, cell pellets for either CK2α or CK2α^Lys198Arg^ plus such for CK2β^1-193^ were merged at a mass ratio of 1:1 and frozen at −80°C. Mixed cells were lysed in 30 ml lysis buffer (500 mM NaCl, 25 mM TRIS/HCl, pH 8.5, 1.3 mg/ml lysozyme, 13.3 μg/ml DNaseI, 2 mM PMSF, 0.5 μg/ml leupeptin, 0.7 μg/ml pepstatin A) and incubated for 30 min at 4°C before subsequent cautious sonification (6 cycles, 20 s on/20 s off, 4°C, 50% power). After centrifugation at 100,000 x g at 4°C for 30 min and filtration, the cleared lysate was applied to a Ni-NTA affinity chromatography column (self-packed, 2 ml). Subsequently, the column was washed first with 5 column volumes of 500 mM NaCl, 25 mM TRIS/HCl, pH 8.5, and then with 10 column volumes of 500 mM NaCl, 25 mM Tris/HCl, pH 8.5, 25 mM imidazole. Finally, bound protein was eluted by addition of 5 column volumes of 500 mM NaCl, 25 mM Tris/HCl, pH 8.5, 250 mM imidazole. Fractions of 1 ml were analysed by SDS-PAGE and those containing the expected CK2α_2_β_2_ holoenzyme variant were pooled and applied to a gel filtration column (Cytiva HiLoad™ 26/600 Superdex™ 200 pg, 320 ml, Thermo Fisher Scientific, Braunschweig, Germany) using an Äkta Start System (GE Healthcare Europe, Freiburg, Germany) to remove further impurities as well as excess CK2 subunits not incorporated in the CK2 holoenzyme. The gel filtration column was equilibrated in 500 mM NaCl, 25 mM Tris/HCl, pH 8.5, serving subsequently as storage buffer. Again, fractions were analyzed by SDS-PAGE and the fractions containing the CK2α_2_β_2_ holoenzyme variants were pooled and stored at −80°C.

Fluorescence-labelled CK2β^1–193^ was prepared to determine the CK2β interaction with either wild-type CK2α or CK2α^Lys198Arg^ by specific fluorescence quenching. For this purpose, the unnatural amino acid *para*-azidophenylalanine (pAzF) purchased from Bachem AG (Bubendorf, Switzerland) was incorporated at position 108 of CK2β^1-193^ following an adapted protocol described before for a CK2α-pAzF construct (Nienberg et al., 2016). The essential modification here was that a CK2β^1–193^ encoding DNA sequence within the plasmid pT7-7 was subjected to site-directed mutagenesis to obtain an amber stop codon at position 108. The resulting plasmid for CK2β^1–193,Tyr108Stop^ and the plasmid pEVOL-pAzF (Chin et al., 2002), which encodes for the amber suppressor tRNA/aminoacyl-tRNA synthetase, were transformed into *E. coli* BL21 (DE3) cells to allow the incorporation of pAzF during translation. The details of the expression procedure were performed as described by Nienberg et al. (2016). Harvested cells were lysed and the lysate was centrifuged at 100,000 x g. The supernatant was filtered using a 0.22 µm filter to remove leftover cell debris and applied to a Ni-NTA affinity chromatography column (self-packed, 2 ml). The column was washed with 20 column volumes of 500 mM NaCl, 25 mM Tris/HCl, pH 8.5, 50 mM imidazole. Finally, CK2β^1–193^-pAzF was eluted by the addition of 5 column volumes of 500 mM NaCl, 25 mM Tris/HCl, pH 8.5, 250 mM imidazole. Fractions of 1 ml were dialyzed against standard storage buffer (500 mM NaCl, 25 mM Tris/HCl, pH 8.5) and analysed by SDS-PAGE. Those containing CK2β^1–193^-pAzF were pooled and labelled with dibenzylcyclooctyne-Sulfo-Cy5 (DBCO-Sulfo-Cy5) in a Strain Promoted Azide-Alkyne Cycloaddition (SPAAC) reaction as described before (Agard et al., 2006; Nienberg et al., 2016).

### Isothermal Titration Calorimetry

The procedure previously described (Raaf et al., 2013) was a adapted to perform ITC measurements. In this work, C-terminally truncated variants of the CK2 subunits were used which previously had been demonstrated to be fully competent to perform the CK2α/CK2β interaction (Raaf et al., 2008). The standard background buffer (500 mM NaCl, 25 mM TRIS/HCl, pH 8.5) was used to dilute the stock solutions of either CK2α^1–335^ or the mutant CK2α^1–335,Lys198Arg^ to a concentration of 7 µM and that of CK2β^1–193^ to 149 µM. The latter was filled into the injection syringe. The measurements were carried out using a VP-ITC (Microcal) at 35°C. In each run, 25 injections of CK2β^1–193^ solution with a volume of 2 µL for the first, and 10 µL for all subsequent injections were applied; the off-time between two single injections was 300 s. Three independent measurements were performed for the CK2α^1–335^/CK2β^1–193^ interaction and two for the CK2α^1–335,Lys198Arg^/CK2β^1–193^ interaction.

The raw ITC data were processed with ORIGIN (version 7), Origin Lab (OriginLab Corporation, Northampton, MA, United States), assuming a binding model of a single set of sites, and with the “ligand is present in the cell” option. For curve fitting, the stoichiometry between CK2α and CK2β was fixed to 1 according to the structure of the CK2α_2_β_2_ holoenzyme (Niefind et al., 2001).

### Fluorescence-Based Assay for Determination of Dissociation Constant

A fluorescence-based assay was used to determine dissociation constants (K_D_ values) for the interaction of either CK2α or CK2α^Lys198Arg^ with CK2β^1–193^-pAzF after coupling the latter with DBCO-Sulfo-Cy5. For detection of fluorescence, a Monolith NT.115 device (Nanotemper, München, Germany) was used. Increasing amounts of either CK2α or CK2α^Lys198Arg^ to give final concentrations from 0.3 nM to 5 µM were mixed to probes of CK2β^1–193^-DBCO-Sulfo-Cy5 with a constant concentration of 30 nM. Samples were dissolved in 500 mM NaCl, 25 mM TRIS/HCl, pH 8.5, 0.05% Tween®20. The fluorescence intensity was determined at 25°C by excitation with a LED-lamp (LED Power: 95%, DBCO-Sulfo-Cy5: λ_abs,max_ = 646 nm; λ_em,max_ = 661 nm). Based on the CK2α- or CK2α^Lys198Arg^-dependent quenching of fluorescence, K_D_ values were determined using the mode “initial fluorescence” in the software MO. Affinity Analysis v2.1.3 (Nanotemper, München, Germany).

### Differential Scanning Fluorimetry

Differential scanning fluorimetry (DSF) (Niesen et al., 2007; Boivin et al., 2013) was applied to probe the effect of the Lys198Arg mutation on the thermal stability of monomeric (unbound) and CK2β-bound CK2α. For these studies, full-length as well as C-terminally truncated versions of CK2α were used together with CK2β^1–193^, the C-terminally truncated form of CK2β (Raaf et al., 2008).

20 µM stock solutions of four different CK2α variants (CK2α, CK2α^Lys198Arg^, CK2α^1–335^, CK2α^1–335,Lys198Arg^) and a 50 µM stock solution of CK2β^1–193^ were prepared. In addition, 3 µL of the commercial dye solution 5,000X SYPRO Orange (Sigma Aldrich) were diluted with 237 µL water. For a DSF scan with an unbound subunit, 2 µL of the diluted dye solution was mixed with 21 µL standard buffer (500 mM NaCl, 25 mM TRIS/HCl, pH 8.5) and with 2 µL stock solution of either CK2β^1–193^ or one of the CK2α variants. To analyze the effect of the CK2α/CK2β interaction on thermostability *via* DSF, 2 µL CK2β^1–193^ stock solution were mixed with 2 µL stock solution of one of the CK2α variants; this mixture was then incubated for 5 min at room temperature and finally supplemented with 19 µL standard buffer and 2 µL diluted SYPRO Orange dye solution.

DSF measurements were performed with the BioRad CFX96™ RT-PCR system using a temperature gradient from 4 to 95 °C with a slope of 1°C per minute, an excitation wavelength of 485 nm, and an emission wavelength of 530 nm. All melting curves were measured as triplicates. Raw data were collected with the CFX Manager™ software (BioRad) and processed with OriginPro 2021 (OriginLab Corporation, Northampton, MA, United States). To determine the inflection points of the direct melting curves (defined as the melting temperatures T_m_), we calculated their first derivatives, modelled the derivative curves with the Gauss-Fit option of OriginPro 2021 and calculated their minima.

### Crystallization and Crystal Structure Determination

Protein crystallization experiments were performed at 20°C using the sitting-drop variant of the vapour diffusion technique. Crystallization conditions for CK2α^Lys198Arg^ and CK2α^1–335,Lys198Arg^ were searched with the “Index Screen” and the “Crystal Screen” collections purchased from Hampton Research. The most attractive crystals were found with the full-length construct CK2α^Lys198Arg^ and the condition G3 of the Index Screen. This combination was selected for subsequent optimization and macroseeding.

The final crystallization droplets were composed of a 1:1 mixture of protein solution (5 mg/ml CK2α^Lys198Arg^ in 500 mM NaCl, 25 mM TRIS/HCl, pH 8.5) and of reservoir solution (0.2 M lithium sulfate, 25% w/v PEG 3350 and 0.1 M Bis-TRIS/HCl, pH 6.5); to these droplets, single crystals from the screening plates were transferred as macro seeds. After equilibration at 20°C, suitable crystals were harvested, shortly transferred to a cryoprotectant mixture composed of 70 µL reservoir solution and 30 µL ethylene glycol, and subsequently vitrified in liquid nitrogen.

X-ray diffraction data of the CK2α^Lys198Arg^ crystals were collected at beamline ID23-2 of the European Synchrotron Radiation Facility (ESRF) in Grenoble (France) equipped with a PILATUS3 X 2M detector. The temperature of data collection was 100 K and the wavelength 0.8731 Å. The raw diffraction data were processed with the AutoPROC pipeline (Vonrhein et al., 2011) which utilized XDS (Kabsch, 2010), POINTLESS and AIMLESS (Evans and Murshudov, 2013) from the CCP4 suite (Winn et al., 2011) and finally STARANISO (Tickle et al., 2018) to improve the data set by anisotropy correction. The structure was solved by molecular replacement with PHASER (McCoy et al., 2007) integrated in the PHENIX package (Adams et al., 2010) and using the CK2α^1–335^ structure with PDB_ID 2PVR (Niefind et al., 2007) as a search model. The refinement was performed with the phenix. refine module (Afonine et al., 2012) of PHENIX (Adams et al., 2010) in combination with COOT (Emsley et al., 2010) for manual corrections.

### Enzyme Kinetics

To determine enzymatic parameters (K_M_, k_cat_, k_cat_/K_M_) of the different enzyme variants, a capillary electrophoresis (CE) based kinase assay was performed (Gratz et al., 2010). Four different CK2 substrate peptides (RRRDDDSDDD, RRRDDDTDDD, RRRDDDSGGD, and RRREDEYDDD) were purchased from GenScript (Leiden, Netherlands). The enzyme reaction rate was determined with these substrates at varying concentrations from 50 to 750 µM. A constant concentration of 500 µM ATP was applied throughout all experiments. Phosphorylation of substrates was performed by addition of CK2α_2_β_2_ holoenzyme variants with a final concentration of either 18.5 nM for (CK2α)_2_(CK2β^1–193^)_2_, if the peptides RRRDDDSDDD or RRRDDDTDDD were the substrates, or 92.4 nM for (CK2α)_2_(CK2β^1–193^)_2_, if the peptides RRRDDDSGGD or RRRDDDYDDD were the substrates, as well as for all reactions with (CK2α^Lys198Arg^)_2_(CK2β^1–193^)_2_. The significantly higher enzyme concentration for some of the reactions was chosen to compensate for low base activity and improve the detection limit. For each setup, the kinase reaction was performed at 37°C and samples for CE were taken at different time points (3, 6, 9, and 12 min). Initial reaction rates were determined by linear regression.

Substrate and product peptides were separated by CE on a ProteomeLab PA800 System (Beckman Coulter, Krefeld, Germany). For instrument control and analysing of the results the 32 karat 9.1 software (Beckman Coulter, Krefeld, Germany) was used. CE was performed with 2 M acetic acid (pH 2) as an electrolyte, a constant current of 30 µA and UV detection at 195 nm.

K_M_ and v_max_ were determined from Lineweaver-Burk diagrams using GraphPad Prism 5 (GraphPad, La Jolla, CA, United States). Finally, turnover numbers k_cat_ and catalytic efficiencies k_cat_/K_M_ were calculated from these values.

## Results and Discussion

### The Lys198Arg Mutation in CK2α Does Not Reduce the Affinity to CK2β

The CK2β interacting region of CK2α is located exclusively at the N-terminal lobe of the kinase domain (Niefind et al., 2001) with Leu41 and Phe54 being the interaction hotspots on the side of CK2α (Raaf et al., 2011). The P+1 loop of CK2α with Lys198 at its center is relatively far away from this region; therefore and because of the conservative nature of the mutation, we did not expect a significant loss of affinity as a consequence of the mutation.

**FIGURE 1:**
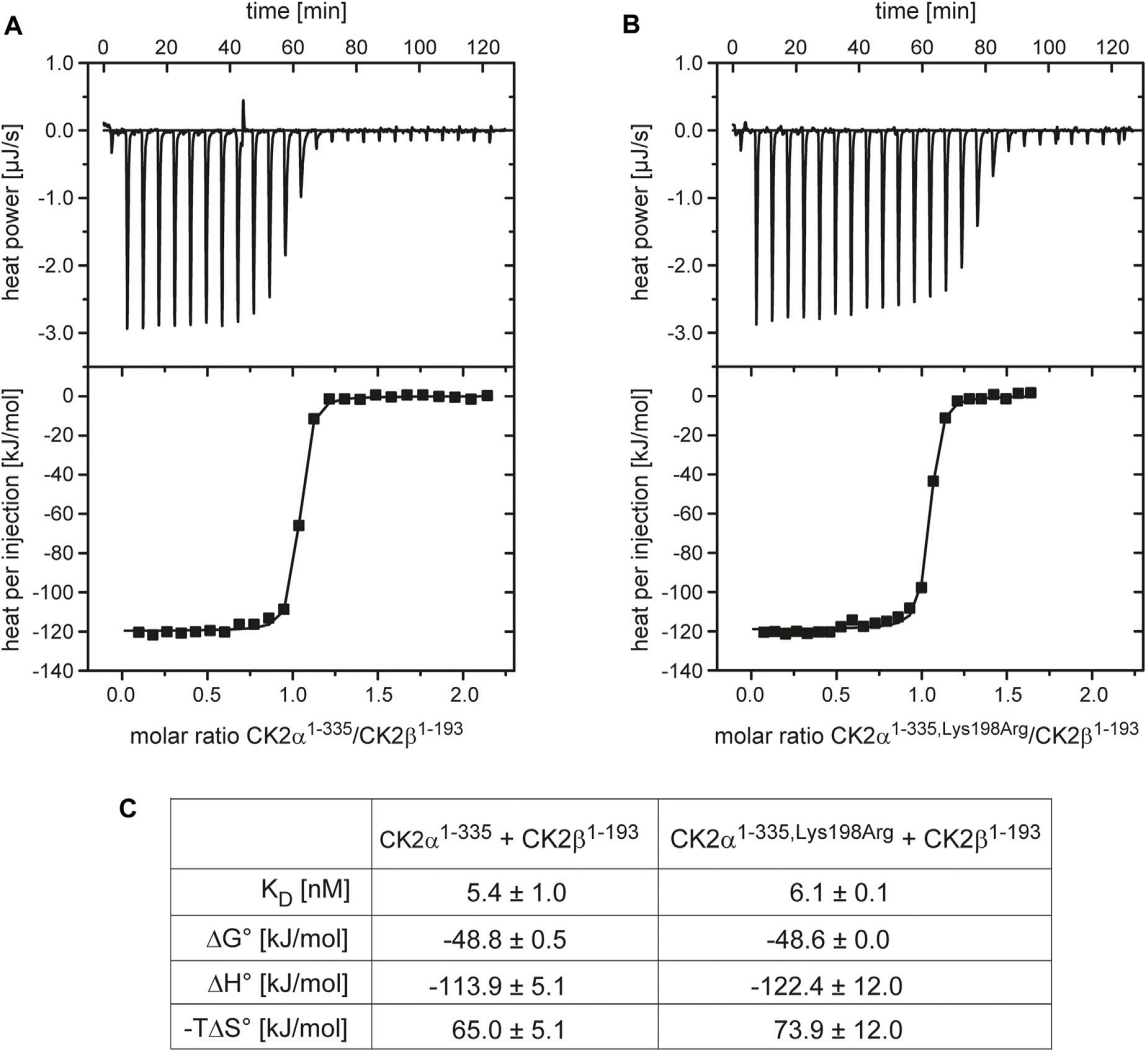
ITC characterization of the effect of the Lys198Arg mutation on the CK2α/CK2β interaction. (A/B) Representative direct and integrated ITC curves for the interaction of CK2β^1-193^ with CK2α^1-335^ **(A)** and CK2α^1-335,Lys198Arg^ **(B)** generated with ORIGIN. **(C)** Thermodynamic data after processing of ITC curves. The numbers represent mean values plus corresponding standard deviations for triplicate measurements.

To probe this quantitatively, we performed ITC experiments, in which CK2β^1–193^ was titrated against either CK2α^1–335^ (Figure 1A) or CK2α^1–335,Lys198Arg^ (Figure 1B), and processed these data to obtain dissociation constants K_D_ plus thermodynamic profiles (Figure 1C). The resulting K_D_ value of 5.4 nM for the CK2α^1–335^/CK2β^1–193^ interaction is in a similar range as reported previously (Raaf et al., 2008; Bischoff et al., 2011; Raaf et al., 2011; Raaf et al., 2013). Significantly, the K_D_ value of the CK2α^1–335,Lys198Arg^/CK2β^1–193^ interaction is nearly identical (6.1 nM). The same is true for the enthalpic and the entropic term of the thermodynamic profile (Figure 1C); in either case, the interaction is driven strongly enthalpically, partially balanced by enthalpy-entropy compensation.

**FIGURE 2:**
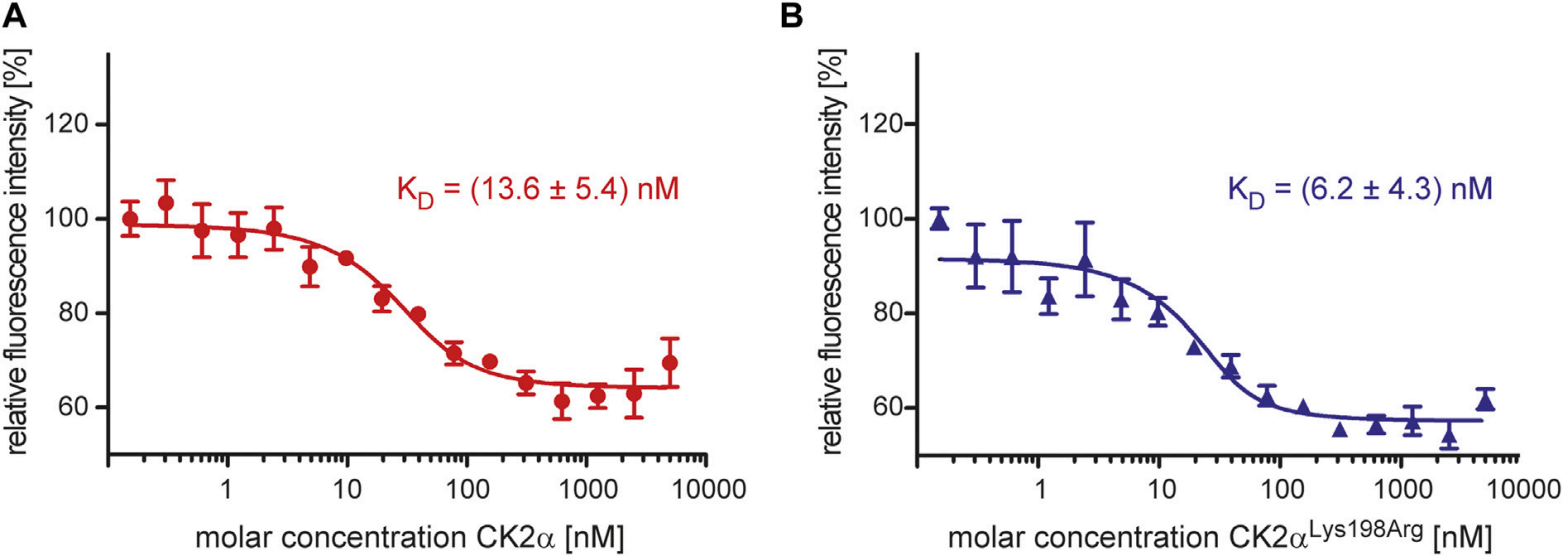
Characterization of the effect of the Lys198Arg mutation on the CK2α/CK2β interaction using a fluorescence-based assay. The fluorescence of CK2β^1-193^-DBCO-Sulfo-Cy5 quenched by increasing concentrations of CK2α **(A)** or CK2α^Lys198Arg^ **(B)** in the range from 0.3 to 5,000 nM was measured. The binding curves were generated and processed using the software MO. Affinity Analysis v2.1.3 (Nanotemper, München, Germany). The K_D_ values given in the graphs are averages from three independent experiments.

To validate these CK2α/CK2β interaction results with an alternative method a fluorescence-based assay was used (Figure 2). Increasing amounts of CK2α, CK2α^Lys198Arg^ or the negative control protein BSA were mixed to fluorescently labelled CK2β^1–193^-DBCO-Sulfo-Cy5. The quenching of fluorescence proved specific binding for CK2α (Figure 2A) and for CK2α^Lys198Arg^ (Figure 2B) to the CK2β construct while for BSA no effect on fluorescence was detected (data not shown). Quantitative processing of these data resulted in K_D_ values of 13.6 nM for the CK2α/CK2β^1–193^-DBCO-Sulfo-Cy5 interaction (Figure 2A) and 6.2 nM for the CK2α^Lys198Arg^/CK2β^1–193^-DBCO-Sulfo-Cy5 interaction (Figure 2B) which were not significantly different from each other (*p* > 0.05, One-way-ANOVA). Furthermore, the K_D_ values are in a similar range as those determined with ITC (Figure 1C) or reported for using Microscale thermophoresis (MST; 12 nM; (Pietsch et al., 2020). This indicates that measuring of fluorescence quenching of CK2β^1–193^-DBCO-Sulfo-Cy5 has the potential to become a novel methods to determine K_D_ values of the interaction of CK2β with variants of CK2α.

In summary, the CK2α/CK2β interaction data determined *via* ITC and fluorescence quenching are largely consistent with the expectation that the CK2α mutation Lys198Arg has no significant effect on the interaction with CK2β.

### The Thermostability of CK2α and of the CK2α_2_β_2_ Holoenzyme Is Not Affected by the Lys198Arg Mutation

**FIGURE 3:**
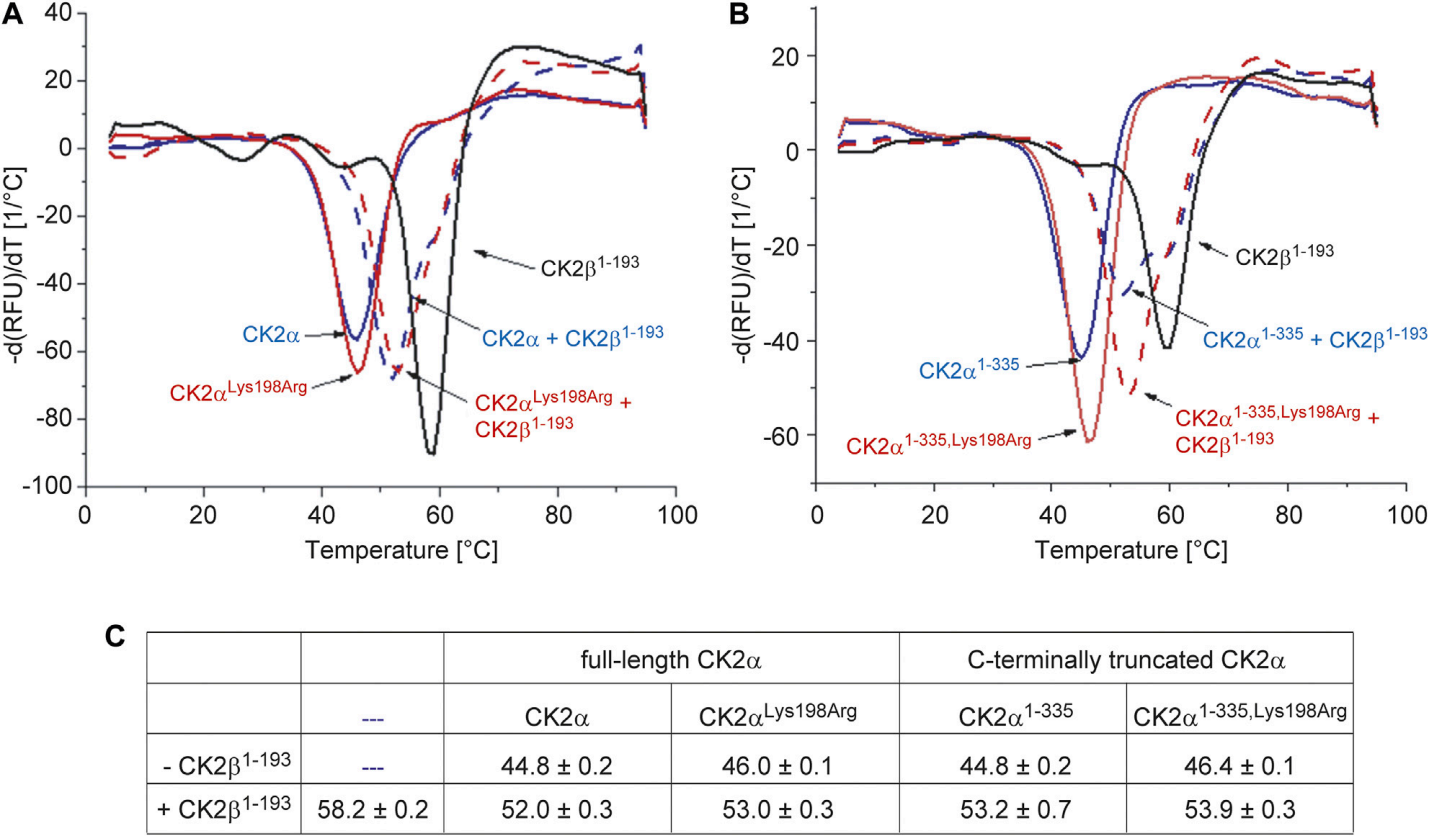
Differential scanning fluorimetry to determine the thermal stability of the CK2α variants of this study and its change by CK2β^1-193^. **(A/B)** Negative first derivatives of representative melting curves of either full-lengths CK2α variants **(A)** or C-terminally truncated CK2α variants **(B)** in the absence of CK2β^1-193^ (solid blue and red lines) and in the presence of CK2β^1–193^ (dashed blue and red lines). For comparison, the curve measured with unbound CK2β^1–193^ is drawn as black solid line in both panels. **(C)** Melting temperatures derived from DSF curves as visible in panels A and B. Averages and standard deviations of three independent measurements, respectively, are given.

CK2β is not only significantly more thermostable than CK2α, but its interaction with the latter has also a strong stabilizing impact against thermal stress (Boldyreff et al., 1993; Raaf et al., 2008). These facts known from differential scanning calorimetry experiments (Raaf et al., 2008) could be confirmed here with DSF (Figure 3): the T_M_ value of CK2β^1–193^ was determined as 58.2°C while the melting temperature of unbound CK2α was almost 14° lower (44.8°C), irrespective if the full-length versions of CK2α were tested (Figure 3A) or the C-terminally truncated constructs (Figure 3B). After binding to CK2β^1–193^, however, the T_M_ values of the CK2α variants increased to 52°C (full-length CK2α) and to 53.2°C (CK2α^1–335^).

A comparable thermostabilization effect by CK2β^1–193^ should exist if—as the ITC (Figure 1) and fluorescence quenching (Figure 3) data suggest—the CK2α mutation Lys198Arg does not impair the assembly of the CK2α_2_β_2_ holoenzyme. In fact, according to the DSF data, the T_M_ value of CK2α^Lys198Arg^ increases from 46°C (unbound) to 53°C (CK2β^1–193^-bound) and the T_M_ value of CK2α^1-335,Lys198Arg^ from 46.4°C (unbound) to 53.9°C (CK2β^1–193^-bound). Thus, the ITC/fluorescence evidence that the Lys198Arg mutation of CK2α does not affect the CK2α/CK2β interaction is emphasized by the DSF results.

Noteworthy, the T_M_ value of CK2α^Lys198Arg^ is about 1° higher than that of wild-type CK2α regardless whether CK2β^1–193^ is bound or not. A similar increase is visible if the C-terminally truncated variants are compared. Thus, the mutation Lys198Arg itself causes a slight, but significant thermostabilization of CK2α, an observation that is consistent with the conservative nature of the mutation.

### The Lys198Arg Mutation in CK2α Causes a Shift of the Anion Binding Site at the P+1 Loop

The structure of CK2α^Lys198Arg^ was solved by molecular replacement and refined to a resolution of 1.77 Å (Table 1). The asymmetric unit of the tetragonal CK2α^Lys198Arg^ crystal contains two protein molecules. While their C-terminal segments were not visible in the electron density, both CK2α^Lys198Arg^ chains were largely well defined from residue 2 to 333 and in particular in the region of the P+1 loop, which contains the mutated position 198.

**TABLE 1:** X-ray data and refinement statistics of a CK2α^Lys198Arg^ structure.

X-ray diffraction data quality	
Wavelength [Å]	0.87313
Synchrotron (beamline)	ESRF (ID23-2)
Space group	P4_3_2_1_2
Unit cell: a, b, c [Å]	127.824, 127.824, 123.180
α, β, γ [°]	90.0, 90.0, 90.0
Protomers per asymmetric unit	2
Resolution [Å] (highest shell)	88.698–1.774 (1.980–1.774)^a^
R_sym_ [%]	58.7 (281.4)^a^
CC1/2	0.990 (0.709)^a^
Signal-to-noise ratio (I/σ_I_)	18.1 (1.8)^a^
No. of unique reflections	65,649 (3,280)^a^
Completeness (spherical) [%]	66.5 (12.0)^a^
Completeness (ellipsoidal) [%]^b^	96.3 (80.1)^a^
Multiplicity	44.4 (38.1)^a^
Wilson B-factor [Å^2^]	3.97
Refinement and structure quality	
No. of reflections for R_work_/R_free_	64,295/1,332
R_work_/R_free_ [%]	18.0/21.8
Number of non-H-atoms	6,395
Protein	5,620
Ligand/ion	84
Water	691
Average B-factor [Å^2^]	20.34
Protein	19.25
Ligand/ion	32.33
water	27.75
RMS deviations	
Bond lengths [Å]	0.004
Bond angles [°]	0.65
Ramachandran plot	
Favoured [%]	98.18
Allowed [%]	1.82
Outliers [%]	0.00

a The values in brackets refer to the highest resolution shell.

b After anisotropic analysis with STARANISO (Tickle et al., 2018).

**FIGURE 4:**
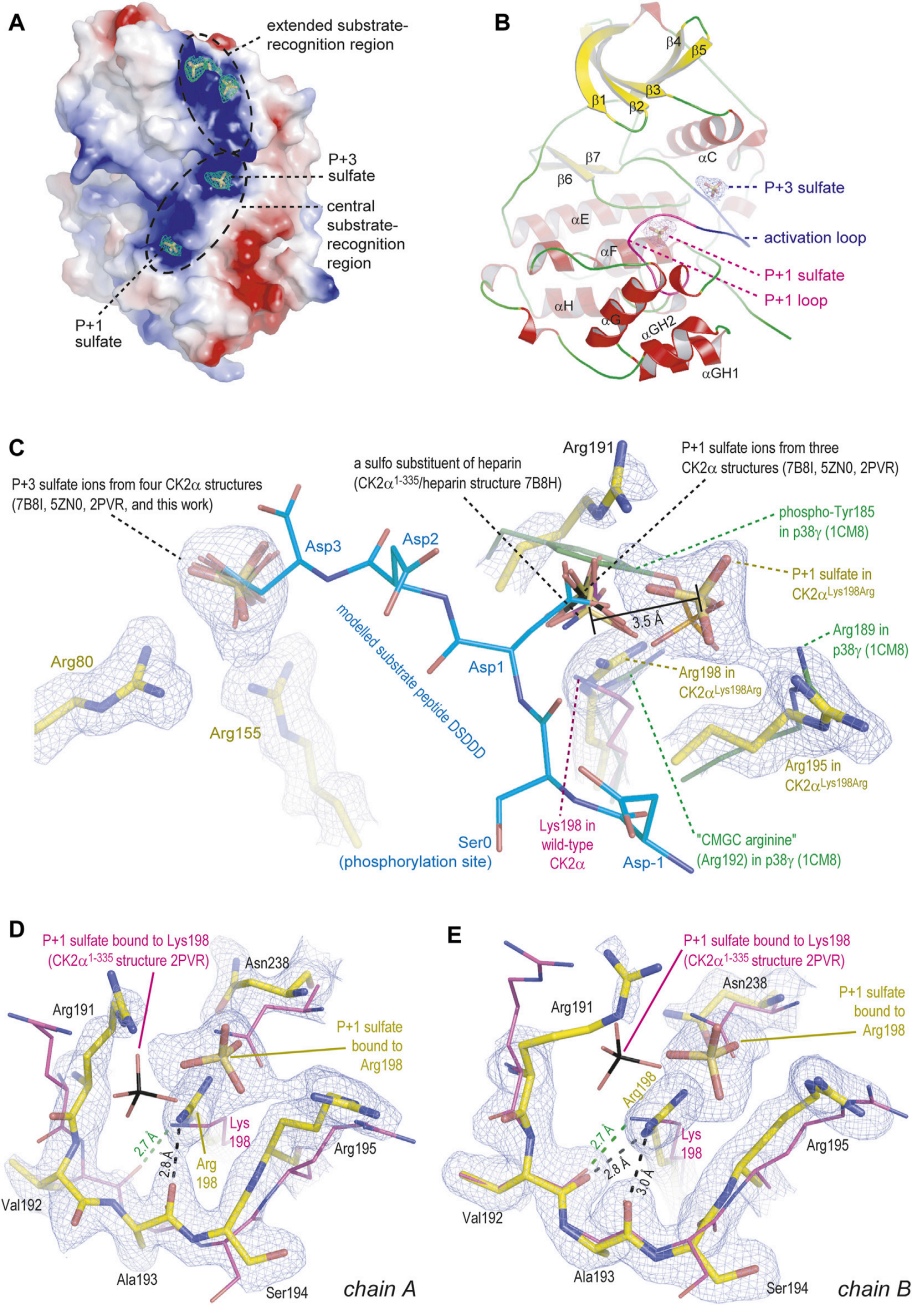
Crystal structure of CK2α^Lys198Arg^. All depicted pieces of electron density were extracted from the final 2F_o_-F_c_ map using a contouring level of 1 σ. The figure was prepared with PyMOL (2013). **(A/B)** Global overview of one CK2α^Lys198Arg^ protomer, illustrating either the electrostatic surface with bound sulfate ions **(A)** or the secondary structure elements **(B)**. **(C)** Details of the anion binding sites at the activation loop (with bound P+3 sulfate) and at the P+1 loop. A substrate peptide modelled into the active site as described by Niefind et al. (2007) is shown with light-blue carbon atoms. Sulfate ions or a heparin sulfo group from several other wild-type-like CK2α structures (Niefind et al., 2007; Shibazaki et al., 2018; Schnitzler and Niefind, 2021) as well as the essential part of the P+1 loop in the MAP kinase p38γ (Bellon et al., 1999) were drawn for comparison. **(D/E)** The P+1 loop in chain A **(D)** or in chain B **(E)** of the CK2α^Lys198Arg^ structure; in both cases, the P+1 loop in the wild-type-like CK2α^1-335^ structure 2PVR (Niefind et al., 2007) plus the bound sulfate ion were drawn to illustrate changes in the backbone and the precise sulfate ion location.

During structure refinement, a number of large and approximately tetrahedrally formed pieces of electron density emerged at positively charged surface areas of the enzyme molecules known to be relevant for substrate recognition. We filled them with sulfate ions because of the relatively high sulfate content in the crystallization solution (200 mM) and the absence of alternative anionic candidates (Figure 4A).

Sulfate ions are substrate-competitive CK2 inhibitors (Niefind et al., 2007). Some of the bound sulfate ions are coordinated by a part of the N-terminal kinase domain that receives its positive charge largely by a CK2α-typical, lysine-rich sequence motif K^74^KKKIKR^80^ located at the beginning of the helix αC (Figure 4B). This surface patch is referred to as “extended substrate-recognition region” in Figure 4A because it supports the binding of acidic substrates as demonstrated by mutational analyses (Sarno et al., 1995; Sarno et al., 1997) and by the identification of an interface to the substrate-competitive inhibitor heparin, a highly sulfated, negatively charged carbohydrate (Schnitzler and Niefind, 2021).

In the context of this study, two sulfate ions located at the “central substrate-recognition region” (Figure 4A) are particularly relevant because their binding sites are the P+1 loop, which harbors the critical Lys198Arg mutation, and the activation loop (Figure 4B). At these two cavities, sulfate ions have been found several times before (Figure 4C) (Niefind et al., 2007; Shibazaki et al., 2018; Schnitzler and Niefind, 2021); in a recent high-resolution structure of a CK2α^1–335^/heparin complex, one of them—the P+1 loop site—even harbors a sulfo moiety of a heparin disulfo-glucosamine residue (Figure 4C) (Schnitzler and Niefind, 2021). The most striking feature of the CK2α^Lys198Arg^/sulfate complex structure is that the position of the sulfate ion at the activation loop, which is designated as “P+3 sulfate” in Figure 4A/B/C for reasons explained below, is identical compared to the wild-type structures while the sulfate ion at the mutated P+1 loop was shifted by 3.5 Å in the direction of Arg195, one of the selectivity determinants of the P+1 loop (Figure 4C). Noteworthy, a similar displacement of the P+1 loop anion binding site was described previously when CK2α was structurally compared with its closest relatives in the CMGC kinase family (Niefind et al., 2007); it is illustrated in Figure 4C for p38γ which like other MAP kinases requires two phosphorylations for activation and harbors the resulting terminal anionic phospho groups at the activation loop and the P+1 loop (see phospho-Tyr185 of p38γ in Figure 4C) (Bellon et al., 1999). Significantly, p38γ such as most CMGC kinases possesses an arginine at the center of the P+1 loop, meaning equivalent to Lys198 of CK2α; this arginine residue is so typical that it was entitled “CMGC arginine” in a comprehensive evolutionary study (Kannan and Neuwald, 2004). Thus, an arginine at the centre of the P+1 loop can be canonically present as in most CMGC kinases or it can be the result of a mutation as in CK2α^Lys198Arg^, but in both cases it shifts the binding site for anionic moieties within the P+1 loop significantly compared to wild-type CK2α.

The relocation of the P+1 sulfate ion is visible in both protomers of the CK2α^Lys198Arg^ structure (Figure 4D/E). It is accompanied by an evasion of the Asn238 side chain and additionally in chain A (but not in chain B) by a flip of the peptide group linking Val192 and Ala193 (Figure 4D). The latter detail was never observed before: normally in CK2α structures, this peptide group is turned in such a way that a close hydrogen bond between the carbonyl O-atom of Val192 and the terminal amino group of Lys198 can be formed (depicted as green dotted line in Figure 4D/E) with the consequence of structural tension in the peptide backbone at Ala193, indicated by an unfavourable φ/ψ-combination in a Ramachandran graph (Niefind et al., 2007), but released by a peptide flip as visible in chain A. Thus, the tendency to turn the Val192/Ala193 peptide to a relaxed conformation leads to less backbone strain in the P+1 loop of CK2α^Lys198Arg^ compared to wild-type CK2α, an observation that fits to the gain of thermostability mentioned above (Figure 3C).

### Relation to Substrate Specificity and Cushing’s Syndrome

The question arises what the relocation of the P+1 sulfate visible in Figures 4C–E could mean in a functional sense. For the wild-type construct CK2α^1-335^, the binding sites for sulfate ions at the P+1 loop and the activation loop were functionally interpreted by Niefind et al. (2007) who modelled—due to the absence of an experimental CK2α/substrate peptide complex structure—a short CK2 substrate peptide (sequence DSDDD) into the active site of CK2α. The structure of the CK2α-relative cyclin-dependent kinase 2 in complex with cyclin A plus a peptide substrate (Brown et al., 1999) served as a template for this *in silico* modelling. Significantly, the side chain carboxylates of Asp1 and Asp3 of the modelled peptide, which represent the positions P+1 and P+3 of typical CK2 substrates (consensus sequence for substrate recognition: S/T-D/E-X-D/E), coincide well with the two sulfate ions as visible in Figure 4C.

If this overlap is compromised as for the P+1 loop of CK2α^Lys198Arg^, no complete loss of function should be expected, but a disturbance of the canonical substrate recognition. The sulfate shift of 3.5 Å illustrated in Figure 4C suggests that CK2α^Lys198Arg^ still favours substrates with an acidic P+1 residue, but that glutamate with its longer side chain should be boosted compared to aspartate. The preliminary data of Caefer et al. (2021), for which bacterial phosphoproteomes were artificially established by the Proteomic Peptide Library (ProPeL) approach of Lubner et al. (2018), indicate a decreased preference of CK2α^Lys198Arg^ for acidic residues at the P+1 position of substrates, but a detailed analysis of the identified phosphopeptide motifs shows a more differentiated picture: 699 motifs found exclusively with wild-type CK2α disclose a pronounced specificity for aspartate (but not glutamate) at the P+1 position. In 373 motifs unique for CK2α^Lys198Arg^, glycine followed at a clear distance by alanine and leucine is the preferred P+1 residue while acidic residues are no longer represented significantly. In a subset of 651 overlapping motifs (found with wild-type CK2α as well as CK2α^Lys198Arg^), aspartate is the most frequent P+1 residue closely followed by glutamate. Insofar, a selectivity shift at the P+1 position of substrates from aspartate to glutamate, which is consistent with our structural data, is indeed visible from the preliminary results of Caefer et al. (2021). Simultaneously, however, the Lys198Arg mutation seems to be accompanied by an overall decrease of P+1 preference for acidic residues and a general loss of relevance of the P+1 loop for substrate recognition. These tendencies are not apparent from the CK2α^Lys198Arg^/sulfate structure presented here. Crystal structures of CK2α and CK2α^Lys198Arg^ in complex with substrate peptides are required in the future to explain these changes of substrate specificity.

Interestingly, the ProPeL method was also applied to investigate the mutation Leu205Arg of protein kinase A (PKA) (Lubner et al., 2017) which is a central driver of Cushing’s syndrome caused by cortisol-secreting adenomas (Cao et al., 2014; Berthon et al., 2015; Walker et al., 2019). Leu205 of PKA is equivalent to Lys198 of CK2α, meaning it is the central residue of the P+1 loop in PKA and one of determinants of the enzyme’s preference for hydrophobic residues like Phe, Leu, Ile or Val at the P+1 position of substrates. For PKA^Leu205Arg^, Lubner et al. (2017) determined still a certain P+1 preference for Leu, but Asp, Asn and Gln emerged as new strongly acceptable P+1 residues in substrate proteins. Thus, again a subtle shift of protein kinase selectivity—caused by a mutation in the P+1 loop—rather than a loss of function seems to be a genetic background of a protein kinase-linked disease.

### Michaelis-Menten Kinetics

**FIGURE 5:**
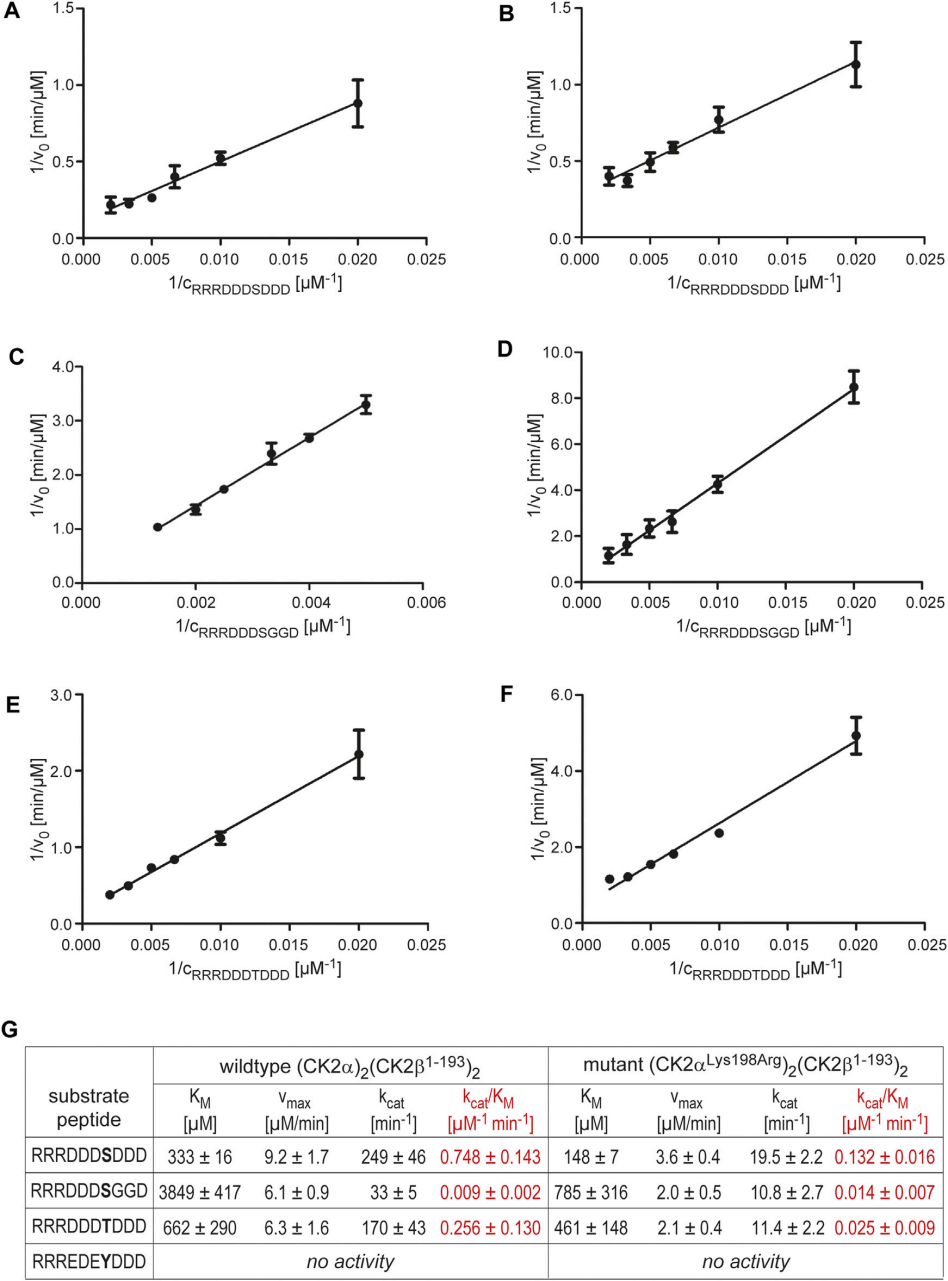
Michaelis-Menten kinetics with varying substrate concentrations to examine changes in substrate specificity caused by the CK2α mutation Lys198Arg. |**(A/C/E)** Lineweaver-Burk plots for wild-type (CK2α)_2_(CK2β)_2_ and the substrates RRRDDDSDDD **(A)**, RRRDDDSGGD **(C)**, or RRRDDDTDDD **(E)**. **(B/D/F)** Lineweaver-Burk plots for the mutant (CK2α^Lys198Arg^)_2_(CK2β)_2_ and the substrates RRRDDDSDDD **(B)**, RRRDDDSGGD **(D)**, or RRRDDDTDDD **(F)**. **(G)** Overview of the enzymological data derived from the Lineweaver-Burk plots in panels **(A–F)**. The catalytic efficiency values (k_cat_/K_M_) are highlighted in red colour.

Potential selectivity changes caused by the Lys198Arg mutation were examined. To this end, we performed comparative Michaelis-Menten kinetics to determine K_M_- and k_cat_-values (and out of them catalytic efficiencies) of CK2α_2_β_2_-holoenzyme complexes with either wild-type CK2α or CK2α^Lys198Arg^ for the phosphorylation of four different peptide substrates (Figure 5). An established CK2 standard substrate with the sequence RRRDDDSDDD served as positive control (Figure 5A/B). The other substrate peptides with sequences RRRDDDSGGD (Figure 5C/D), RRRDDDTDDD (Figure 5E/F), and RRREDEYDDD were inspired by the preliminary results of Caefer et al. (2021). With CK2α^Lys198Arg^, these authors had observed selectivity changes at the P+1 position as mentioned above and a decreased preference for Thr phosphorylation, but an increased propensity for phosphorylation at Tyr, in particular if the Tyr phosphorylation site is preceded by acidic residues at the P-2 and the P-1 position.

With the peptide RRREDEYDDD, we could not detect any significant tyrosine phosphorylation, either with the wild-type CK2α_2_β_2_ holoenzyme or with the mutant (CK2α^Lys198Arg^)_2_(CK2β)_2_. Thus, no conclusions concerning changes in phosphoacceptor specificity and no comparison with the corresponding preliminary results of Caefer et al. (2021) can be drawn. Possible reasons for this failure are the nature of the peptide or the limited sensitivity of the assay read-out (or a combination of both). According to an early *in vitro* study by Marin et al. (1999) with various substrates peptides, the Tyr phosphorylation of CK2 depends extremely on the sequence environment; for instance, the peptides RRRADDYDDDDD, EEEEEYEEEEEEE, and PEGDYEEELE remained completely unphosphorylated in spite of acidic residues at the P+1 and P+3 positions, and even for peptides with detectable Tyr phosphorylation by CK2, the catalytic efficiencies for the phosphorylation of equivalent Ser peptides were higher by a factor of at least 10,000 (Marin et al., 1999).

In contrast to an increased propensity for Tyr phosphorylation, other tendencies mentioned in the preliminary report of Caefer et al. (2021) are confirmed by the enzymological data summarized in Figure 5G. For the standard peptide RRRDDDSDDD (Figure 5A/B), the Lys198Arg mutation decreased the catalytic efficiency by a factor of 5.7, but for Thr phosphorylation (peptide RRRDDDTDDD; Figure 5E/F), the loss of catalytic efficiency is even higher (factor 10.2). Thus, the phosphoacceptor propensity of Thr compared to Ser is reduced by the Lys198Arg mutation as found by Caefer et al. (2021).

Likewise, the catalytic efficiencies we determined for the peptide RRRDDDSGGD (Figure 5C/D/G) are consistent with the preliminary data of Caefer et al. (2021). As mentioned above, these authors reported an increase of phosphopeptide motifs with glycine at the P+1 position when CK2α^Lys198Arg^ rather than the wild-type was used to generate a bacterial phosphoproteome. In line with this, we observed no loss of catalytic efficiency by the Lys198Arg mutation for peptide RRRDDDSGGD in contrast to RRRDDDSDDD, but even a slight, albeit statistically not significant increase from 0.009 μM^−1^ min^−1^ to 0.014 μM^−1^ min^−1^ (factor 1.6). This shows that glycine is in fact a preferred P+1 residue in substrates of CK2α^Lys198Arg^, and it supports the notion of Caefer et al. (2021) that CK2α^Lys198Arg^ might have decidedly new and unique substrate proteins not phosphorylated under the catalysis of wild-type CK2.

## Conclusion

In summary, the enzymological data of this work fit to its central structural finding that the Lys198Arg mutation causes a shift of the anion binding site at the P+1 loop. Furthermore, they support the conclusion drawn in the preliminary report of Caefer et al. (2021) that the CK2α mutant Lys198Arg does not primarily lead to a loss of function, but to alterations of the phosphoacceptor preference and the substrate specificity. Taylor et al. (2012) emphasized that EPKs principally differ from metabolic enzymes because they are not evolutionarily optimized for substrate turnover and because the physiological concentrations of their protein substrates are typically low and often in the same range as the enzyme concentrations themselves. Insofar, the general reduction of the catalytic activity of an EPK is perhaps less detrimental than subtle changes in substrate specificity which disturb regulatory networks. Since OCNDS is a neurodevelopmental disease and Lys198Arg the most frequent OCNDS mutation (Nakashima et al., 2019), specific proteins of the nervous system are probably differentially phosphorylated by CK2α and the mutant CK2α^Lys198Arg^. Caefer et al. (2021) predicted a number of ion channels localized in the axon of neurons as candidates for such a differential phosphorylation. It remains to be shown if these predictions are valid, which other changes of the CK2-dependent phosphoproteome (in particular in neurons) are caused by the mutation Lys198Arg and how these are linked to neurodevelopmental processes leading to the OCNDS phenotype. Such an understanding of the molecular basis of OCDNS may finally result in translational approaches and therapies.

## Data Availability

The crystallographic data (atomic coordinates and structure factors) of the CK2α^Lys198Arg^/sulfate complex structure are available from the Protein Data Bank (PDB) under the accession code 7PSU : https://doi.org/10.2210/pdb7PSU/pdb.
